# Exposure to cell phone radiofrequency changes corticotrophin hormone levels and histology of the brain and adrenal glands in male Wistar rat

**DOI:** 10.22038/ijbms.2018.29567.7133

**Published:** 2018-12

**Authors:** Sima Shahabi, Iman Hassanzadeh Taji, Maedeh Hoseinnezhaddarzi, Fateme Mousavi, Shermineh Shirchi, Atena Nazari, Hooman Zarei, Fereshteh Pourabdolhossein

**Affiliations:** 1 Physiology Department, Faculty of Medicine, Babol University of Medical Sciences, Babol, Iran; 2 Neuroscience Research Center, Health Research Institute, Babol University of Medical Sciences, Babol, Iran; 3 Student Research Committee, Babol University of Medical Sciences, Babol, Iran; 4 Anatomy Department, Faculty of Medicine, Mazandaran University of Medical Sciences, Sari, Iran; 5 Cellular and Molecular Biology Research Center, Health Research Institute, Babol University of Medical Sciences, Babol, Iran

**Keywords:** Adrenal glands, Cell phone, Corticotropin hormone, Cortisol, Hypertrophy, Radio-frequency

## Abstract

**Objective(s):**

Nowadays, the electromagnetic field-emitting devices are used routinely in our lives. Controversial reports exist concerning the effects of mobile radiofrequency (RF) on different parts of the body, especially stress hormones. The main goal of the present work was to study the long-term effects of mobile RF900 MHz exposure with special focus on the adrenal gland pathophysiology and function.

**Materials and Methods:**

Adult male Wistar rats were exposed to mobile RF 6 hr daily for 4–8 weeks. Intact and switched-off exposed animals were considered as controls. Plasma ACTH and cortisol levels were measured by the ELISA method. At the end of the experiment, a histological study was done on adrenal gland and brain tissues by hematoxylin and eosin staining. The thickness of the fasciculate layer of the adrenal gland, and its cell count and perimeter were measured using the Fiji software.

**Results:**

Enhanced plasma ACTH and cortisol levels were found after prolonged exposure to mobile RF. The fasciculata layer of adrenal cortex eventually thickened following mobile RF radiation. While the number of cells in zona fasciculata remained constant, the cell size and perimeter increased during RF exposure. Finally, we found that vacuolization in brain tissue and the number and size of vacuoles considerably increased during two months of RF exposure.

**Conclusion:**

Cell phone RF exposure induced significant hormonal and structural changes in adrenal gland and brain tissues. Therefore, the public should be aware and limit their exposure as much as possible.

## Introduction

Nowadays, the electromagnetic field (EMF)-generating equipment like mobile phones, tablets, and other electrical devices have been used in our daily routine lives. The potential adverse health effects of EMF have led to considerable public concern (1). Cell phone radiofrequency (RF) can be different according to the operating system, ranging from 900 to 1800 MHz [Global system for mobile communication (GSM)] and 2200 MHz [Universal mobile telecommunication system (UMTS)] (2). The safe levels of RF of electromagnetic power have been specified in the range of 3 kHz to 300 GHz by The Federal Communications Commission in the United States (FCC) and International Commission on Non-Ionizing Radiation Protection in Europe and some other countries (ICNIRP) (3). The intensity and duration of RF exposure strongly influences its biological effects (4). 

A number of studies have reported the adverse effects of RF radiation on different regions of human and experimental animal bodies (5, 6) such as the central nervous system (3, 7, 8), endocrine system (6, 9-11), liver (12), kidney (13), male and female reproductive systems (14, 15), blood cells, and immune function (9). The mechanism of mobile phone-related biological effects is not known very well; however, the possible reasons are the thermal and non-thermal effects that might disturb the cell function (16). EMF is also considered a stress factor because of its high degree of tissue damage (17).

The cells in the adrenal gland cortex in the fasciculate layer synthesize glucocorticoids (cortisol) in response to adrenocorticotropin hormone (ACTH) from the pituitary gland. This final product of the hypothalamus-pituitary-adrenal (HPA) axis has a protective role during stressful conditions (18). Activation of the HPA axis is an essential part of stress adaptation and survival (19). The effect of EMF exposure on adrenal corticoid response was first studied in the early 1960s (20). Different studies have suggested that EMF exposure may act as a chronic stressor (19, 21). Over the past decades, researchers have revealed that extremely low-frequency magnetic field (ELF-MF) can act on the anxiety-related behavior of animals and the emotional state of people (6, 22, 23). The effects of EMF on stress hormones, depression-like states, and anxiety-related behaviors have been investigated in many studies, and their results are contradictory probably due to differences in the populations, exposure states, and parameters studied (6, 11, 19, 24-26). Some reports stated that long-term ELF-EMF stimulation increases the plasma ACTH and cortisol levels (6, 27); however, another study showed that chronic ELF-EMF exposure did not change or significantly decreased the level of both hormones (19, 28). Animal experiments showed that EMF exposure under different conditions changed the level of cortisol and ACTH and enhanced stress-related behaviors; however, quantitative study of the effects of mobile RF on the adrenal gland structure and HPA axis hormone levels has received little attention. Therefore, the present study was designed to examine the probable effects of chronic exposure of cell phone RF on adrenal gland structure and level of ACTH and cortisol. We also did a histological study on the brain tissue. 

## Materials and Methods

***Animals***

Thirty-two adult male Wistar rats (200–250 g) were obtained from the Animals Breeding and Experimental Research Center of Babol University of Medical Sciences. Eight rats were used in each experimental group. Animals were kept in cages (four rat/cage) with 12 hr light/dark conditions (lights on 07:00 am) and had free access to food and water at the appropriate temperature (23 ± 2 ^°^C). The experimental procedures were designed based on international guidelines for animal studies and approved by the Ethical Committee of Babol University of Medical Sciences. 

***Experimental procedure***

**Figure 1:**
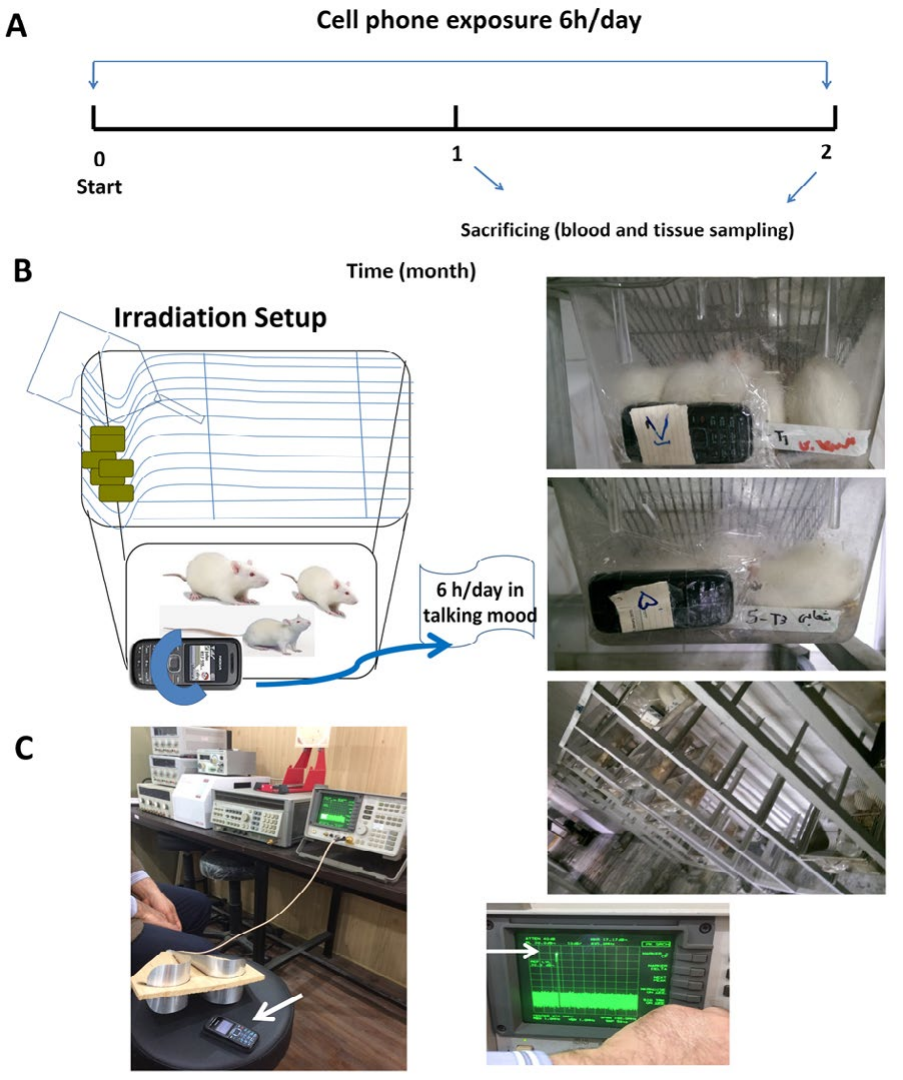
(A) The timeline of the experiment as described in the text. (B) schematic image of irradiation setup used in this study. (C) Measurement of the mobile output by a Spectrum analyzer (HP/ Hewlett Packard-8562A, USA)

Male Wistar rats were randomly divided into four groups (C, SO, E1, and E2) of eight rats each. In the beginning and end of the study, the animals were weighed by an electronic scale with 0.1 g accuracy (Hand®, NS5B). The cell phone model used in our study was Nokia 1208 (China), with personal communications service code division multiple access (PCS CDMA) frequency band of 2G network (900 MHz) and specific absorption rate (SAR) of 1.010 W/kg. Group C was set as a control group, did not receive mobile RF, and was kept under normal conditions. Group SO was also considered as a control group and animals were exposed to switched-off cell phones 6 hr per day for two months. In Group E1, animals received cell phone RF exposure (6 hr per day) for one month. In Group E2, animals received cell phone RF exposure (6 hr per day) for two months. The exposure time was from 9:00 am to 3:00 pm for one to two months. The two control groups were kept in different rooms. The cell phone was placed beside the cage at a distance of 0.5 cm (29) and put in the talking mode, receiving calls from another phone (silent mode) during the exposure time. The cell phone output into the rats’ boxes was measured by a Spectrum analyzer (HP/Hewlett Packard-8562A, USA) in dBm (+15 dBm) and converted to Watt according to (P(mW) = 1 mW. 10(P(dBm)/ 10) formula and was around 31.62 mW. Figure 1 represents the timeline of experiments and a schematic image of the irradiation setup used for the animals.

***ACTH and cortisol assessment***

ACTH and cortisol concentrations were measured by an ELISA method. At the end of experiments, blood samples (2 ml) were collected by the retro-orbital puncture method at the same time (at 4 pm) for all experimental groups. Samples were added into micro-tubes with 5% EDTA on ice and immediately centrifuged at 4 ^°^C in 5000 rpm for 10 min. The separated plasma was kept at -80 ^°^C until analysis. The ELISA assay measured ACTH and cortisol levels by using the appropriate kits (CUSABIO CO., Japan). All samples were assayed in duplicate. The sensitivity of rat ACTH ELISA kit (code: CSB-E06875r) was 1.25 pg/ml with detection range from 1.25 pg/ml to 50 pg/ml. The sensitivity of rat cortisol ELISA kit (code: CSB-E05112r) was 0.049 ng/ml with detection range from 0.049 ng/ml to 200 ng/ml.

***Histological study***

At the end of experiments, the rats were euthanized under ketamine (70 mg/kg) and xylazine (10 mg/kg) anesthesia and perfused with 0.1 M PBS followed by 4% paraformaldehyde in 0.1 M PBS (pH 7.4). Adrenal gland and brain tissues were removed and post-fixed overnight at 4 ^°^C in PFA 4%. After dehydration with different gradients of alcohol, tissues were cleared by incubation in xylene and finally embedded in paraffin and blocked. Serial coronal sections (6 μm) were obtained using a microtome (Leica, Germany). Paraffin-embedded sections were used for H&E staining. Images were captured with a DP-27 camera from the adrenal glands and cerebral cortex. Histological data were analyzed by a pathologist blinded to the experimental groups. The thickness of the fasciculate layer of the adrenal gland, its cell count, and perimeter were measured using Fiji software.

***Statistical analysis***

The results are expressed as mean±SEM. The data were analyzed by one-way analysis of variance (ANOVA) followed by Tukey’s *post hoc* using Graph Pad PRISM software (Graph Pad software, Inc, San Diego. CA). Data were determined to be significant when *P*<0.05.

## Results

***Mobile phone RF 900 MHz triggers ACTH and cortisol secretions***

**Figure 2:**
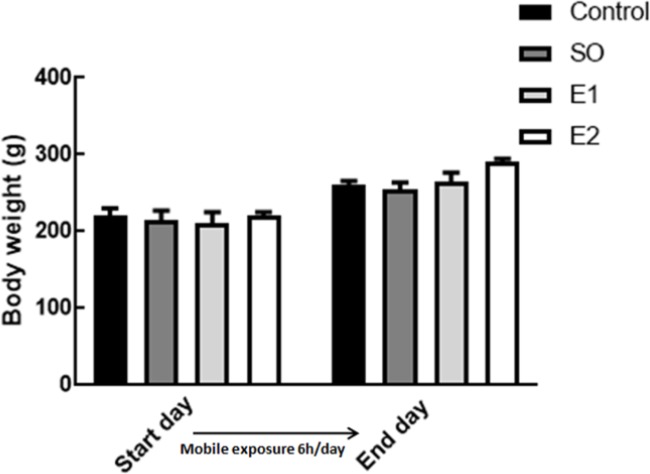
The effect of chronic exposure of mobile phone radiation on body weight. There were no significant changes between the groups in initial or final body weight. C: Control group, SO: switched-off mobile exposed group (6 hr/day), E1: Mobile RF exposed group (6 hr/day) for one month, E2: Mobile RF exposed group (6 hr/day) for two months. Data are expressed as mean±SEM of eight animals per group

**Figure 3:**
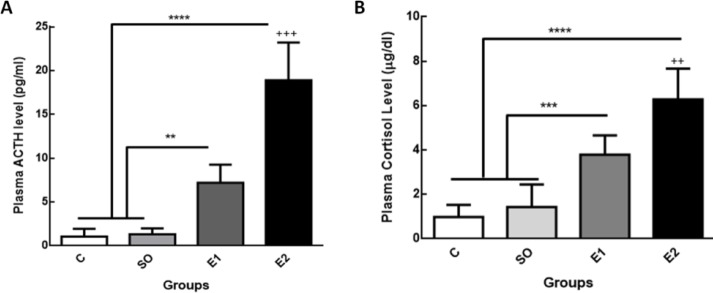
The effect of mobile phone radiation (900 MHz) on plasma levels of ACTH and cortisol in rats. (A) Mobile phone radiation (6 hr/day) for the duration of one (E1) and two (E2) months significantly increased ACTH levels compared to control (C) and switched-off mobile phone exposed groups (SO) (**P<0.01, ****P<0.0001, respectively). The ACTH level was significantly enhanced in E2 compared to the E1 group (+++*P*<0.001). (B) Cortisol concentration in E1 and E2 groups considerably increased compared to control and SO groups (***P<0.001, ****P<0.0001, respectively). In E2 cortisol level was notably raised compared to the E1 group (++*P*<0.01). Data are expressed as mean±SEM of eight animals per each group

Comparison of the animal initial and final body weight between groups revealed that chronic exposure to mobile phone radiation has no effect on animal body weight, although during the experimental period all animals gained weight independent of mobile exposure (Figure 2). Our results showed that plasma levels of ACTH in rats exposed to cell phone RF 900 MHz (6 hr/day) for one and two months significantly increased compared to control groups (intact and switched-off mobile exposed animals) (Figure 3A,^**^P<0.01, ^****^P<0.0001, respectively). The time course of RF exposure also affected the ACTH level indicating that the ACTH level after two-month exposure to mobile RF was considerably enhanced in comparison with one month (Figure 3 A,^+++^ *P*<0.001). The cortisol concentration also drastically increased in one (E1) and two (E2) month cell phone RF exposure groups compared to the controls, respectively (Figure 3B,^ ***^P<0.001, ^****^P<0.0001) with the E2 group experiencing higher cortisol levels compared to the E1 group (Figure 3 B,^++^*P*<0.01).

***Long-term exposure to cell phone RF induces hypertrophy and disorganization of zona fasciculata of the adrenal gland***

**Figure 4:**
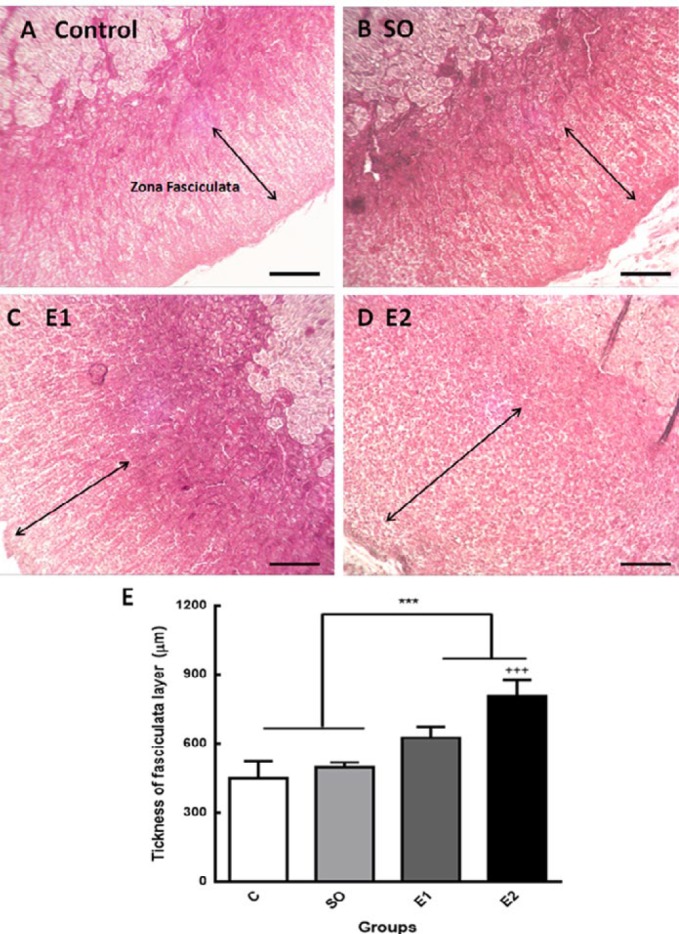
The thickness of the fasciculata layer of adrenal glands enhanced following exposure to mobile radiation. Adrenal gland tissue section (6 μm) stained with hematoxylin and eosin (H&E) in the control group (A), switched-off mobile exposed animals (B), mobile phone radiofrequency (RF) exposed (6 hr/day) animals for one month (C), and mobile phone RF exposed ( 6 hr /day) animals for two months (D). Zona Fasciculata (ZF) thickness was measured with Fiji (Image J) software. (E) Statistical analysis showed that ZF thickness in mobile RF exposed animals (E1 and E2) was significantly increased compared to Control and SO groups (***P<0.001). ZF thickness in the E2 group considerably increased compared to the E1 group (+++*P*<0.001). Arrows show the ZF thickness. Scale bars are 200 µm

The zona fasciculata (ZF) of the adrenal glands is located between the zona glomerulosa and zona reticularis and its cells have clear, lipid-rich cytoplasm and are arranged in radial cords. This layer secretes mainly cortisol in response to ACTH from the anterior pituitary (30). In this study, the thickness of ZF, its cell count, and perimeter in different groups were measured. Our data indicated that one and two-months exposure to cell phone RF considerably increased the thickness of fasciculate layer compared to control animals (Figure 4, A-E, ^***^P<0.001). The two-month exposure to mobile RF remarkably increased the thickness of ZF compared to the one-month exposed group (E1), which indicated that the time course of RF exposure is an effective variable (Figure 4 E, ^+++^*P*<0.001). The thickness of the other two layers of adrenal cortex was not significantly changed in response to mobile RF exposure. This result indicates that mobile RF exposure provokes hypertrophy of the fasciculate layer in the adrenal cortex.

**Figure 5:**
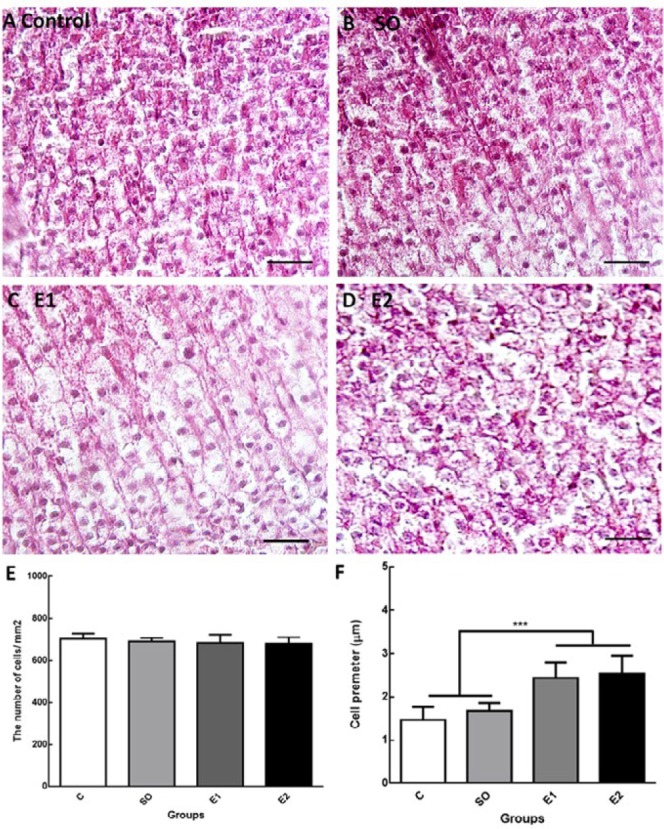
Adrenal gland hypertrophy following exposure to mobile radiation. H&E staining of adrenal gland tissue section (6 μm) in controls (A), switched-off mobile exposed animals (SO) (B), mobile phone radiofrequency (RF) exposed (6 hr /day) animals for one month (E1) (C), and mobile phone RF exposed (6 hr/day) animals for two months (E2) (D). The zona fasciculata (ZF) cell count and perimeter was analyzed with Fiji (Image J) software. (E) Statistical analysis showed that RF exposure did not significantly change the number of cells in ZF of the adrenal glands in different groups. (F) The perimeter of ZF cells considerably increased in E1 and E2 groups compared to control and SO groups (***P<0.001). There is no significant change in cell size between E1 and E2 groups; however, ZF radial cord arrangement was disturbed in E2 compared to E1 and other groups. Scale bars are 50 µm

Next, we counted the cell count of ZF per mm^2^ and no differences were found in different conditions (Figure 5 A-E). However, quantification of cells size and perimeter in the fasciculate layer demonstrated that one and two-month exposure to mobile RF (6 hr/day) significantly increased the cell perimeter relative to the control groups (Figure 5F, ^***^P<0.001). There was no significant change in cell perimeters between one (E1) and two-month (E2) exposed groups (Figure 5F). The constituent cells were arranged into bundles in the fasciculate layer of the adrenal glands in control animals. Although the cell organization of ZF was not changed one month following RF exposure, the columnar organization of ZF cells was disoriented two months after RF exposure (6 hr/day) compared to other conditions (Figure 5D). 

***Long-term exposure to cell phone RF (900 MHz) causes vacuolation in brain tissue***

**Figure 6:**
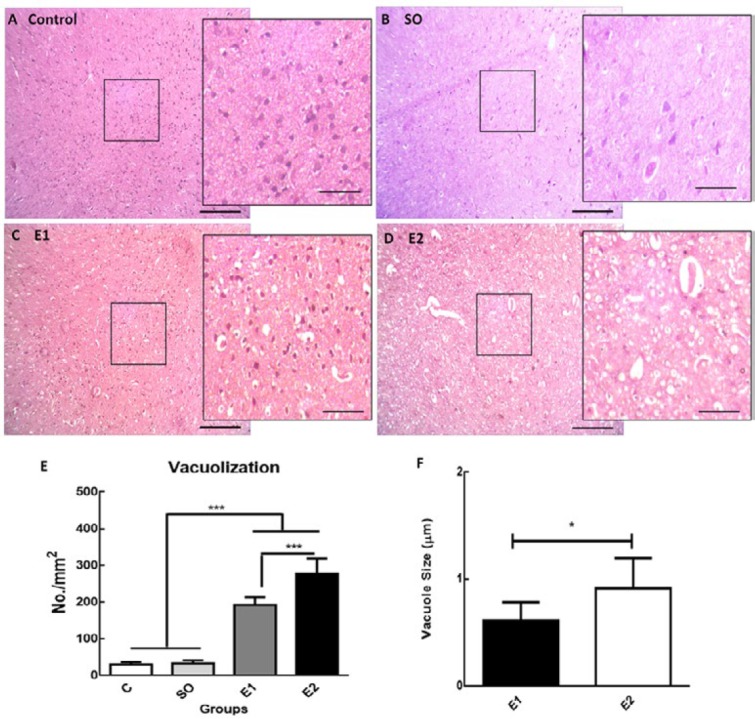
Long-term exposure to mobile RF induces the appearance of vacuoles in brain tissue

The vacuoles within the brain tissue exist as single or grouped with oval to round pale spaces. Cell phone RF exposure 6 hr per day for one and two months resulted in the appearance of more vacuoles with bigger size in the brain tissue (Figure 6 A-D). The vacuole numbers per mm^2^ of brain tissue were calculated using the Fiji software. Microscopic evaluation of the brain sections from control animals (intact and switched-off mobile exposed) demonstrated fewer vacuoles; however, long-term exposure to mobile RF induced prominent vacuoles in multiple brain regions especially in the cerebral cortex (Figure 6 A-D). Vacuole severity (number and size) was greater in animals exposed for longer periods of time. Statistical analysis showed that two-month RF exposure not only significantly enhanced the number of vacuoles (Figure 6 E, *P*<0.001) but also increased the size of vacuoles (Figure 6 F, *P*<0.05) compared to the one-month RF exposed group. However, the number of vacuoles considerably increased following one and two-month RF exposure compared to the control groups (Figure 6 E, *P*<0.001).

## Discussion

The present study indicated that long-term exposure to mobile RF 900 MHz elevated plasma ACTH and cortisol in rats over time. The duration of RF exposures significantly affected the plasma levels of hormones. We also showed that the mobile RF can alter some morphometric and structural parameters of adrenal glands. The thickness of the fasciculata layer was significantly enlarged in RF exposed animals compared to controls. While the cell count did not change in this layer, the cell size and perimeter considerably increased in animals exposed to mobile RF for the duration of one and two months. We also found the widespread vacuolation in brain tissue samples in rats following a chronic exposure to cell phone RF radiation over time. 

H&E staining was used to assess the brain tissue (6 μm sections) architecture and morphology. Vacuoles are the bright spaces of variable size in the brain parenchyma analyzed by Fiji (Image J) software (no/mm2). (A) Brain tissue section from control animal demonstrates normal structure. (B) Brain tissue from animals exposed to a switched-off mobile phone (SO) also indicates normal formation. (C) Exposure to mobile RF (6 hr/day) for one month (E1) induces vacuolization in brain tissue diffusely in the parenchyma. (D) Two-month exposure to mobile RF (6 hr /day-E2) shows increased progressive vacuolation in number and size. (E) Statistical analysis showed that the number of vacuoles per mm2 of brain sections significantly increased in E1 and E2 groups compared to control and SO groups (***P<0.001). Moreover, the number of vacuoles considerably enhanced in E2 compared to the E1 group (+++*P*<0.001). Square boxes display a certain location in the image at high magnification. Scale bars in panels A–D were 200 μm and in their square box they were 40 μm

Nowadays, stress-induced psychopathology is a major health concern and diverse studies have proposed that the EMF exposure can act as a chronic stressor (19, 21). The main aspect of the stress reaction is the activation of the HPA axis, which leads to the rise of ACTH and cortisol (31). In the present study, compatible with other works (6, 24, 27, 32) the levels of ACTH and cortisol were augmented in RF exposed animals over time. Ahmadi Tameh and colleagues showed that long-term exposure to mobile RF increased the cortisol levels in adult rats (32). Another study demonstrated that the plasma ACTH concentration is augmented in the animals after the prolonged exposure to ELF electromagnetic waves (6). ACTH promotes the growth and enlargement of the adrenal cortex and stimulates the secretion of cortisol (33). We can state that the rise of plasma ACTH concentration after prolonged mobile RF exposure promotes adrenal gland activity and increases the cortisol level and thickens the adrenal gland fasciculata layer. However, contrary to our results some other studies revealed that EMF exposure did not change the levels of cortisol or ACTH (19, 34, 35), or reduced the levels of these hormones (25, 26, 36, 37). The reasons for such contradictory results are probably due to differences in the populations, frequency of EMF, exposure states, and parameters studied. For example, Zare and colleagues showed that five-day exposure to ELF-EMF (5–50 Hz) 2 hr/day significantly decreased the cortisol level in the serum of adult guinea pigs (26) and Radon et. al. reported no effects of pulsed radio-frequency electromagnetic fields on cortisol levels in humans (35).

We also confirmed that chronic exposure of the mobile RF can alter the thickness of the fasciculata layer of the adrenal glands. The possible different reasons for the thickened ZF are hyperplasia and hypertrophy (11). We found that the cell count in ZF of adrenal glands did not change upon RF radiation; therefore, the idea of hyperplasia is ruled out. However, we found that the perimeter of the cell in the ZF layer was considerably enlarged in the RF-exposed groups, an observation that confirms the occurrence of hypertrophy (11, 38). Regarding the adrenal cortex hypertrophy under mobile RF exposure conditions, a study revealed that 1 hr daily exposure to 900 MHz mobile EMF for 15 days significantly increased the volume of adrenal zona fasciculate compared to other zones (11). In addition, our results showed that mobile RF exposure led to the structural disorientation of cells in the fasciculata layer of the adrenal cortex only in the two-month exposed group but these changes did not affect the cortisol production by the adrenal glands in these animals. Our data are well-matched with the previous studies regarding the effects of EMF exposure on pathological changes in the adrenal glands (11, 39). An increase in the ZF volume in stressful situations has also been reported (38, 40). Furthermore, numerous studies have confirmed that cell volumes and the mitochondria numbers increased after exposure to EMF (41). It must be noted that most of the reports on effects of EMF exposure on hormones and adrenal glands focus on various ELF-EMF exposures but concerning mobile RF 900–1800 MHz, there are few works conducted on adrenal gland histopathology. 

The adverse effects of EMF on human and experimental animals have been extensively investigated over the past decades and particular interest has been given to the effects of cell phone RF exposure on the central nervous system because of its use in close vicinity to the human brain (42). The effects of mobile RF radiation on the brain, *in vitro* and *in vivo*, from molecular to behavioral and cognitive levels have been repeatedly studied. Hence, we only analyzed the brain tissue in our study to look for any new changes. Quite interestingly, we observed vacuoles in the brain tissue after exposure to mobile RF (6 hr/day) for the duration of one to two months. Exposure for a longer time increased the number and size of the vacuole in the brain parenchyma although it was not associated with neurological dysfunction. Brain vacuolation is a common histopathologic finding that may result from several potential mechanisms related to spongiform encephalopathies, excitotoxicity, and prion diseases (43). However, the pathogenesis of the vacuolation is not clear yet. We did not assess the clinical signs and mechanisms of brain vacuolation in a longer period of time in the present study, which can be carried out in the future.

## Conclusion

Our results proved that exposure to mobile RF significantly enhances the level of ACTH and cortisol hormones over time. Our stereological data confirmed hypertrophy of the fasciculata layer of the adrenal cortex and vacuolization in brain tissue following long-term mobile RF radiation. Finally, cell phone RF exposure induced significant morphological and structural changes in adrenal gland and brain tissues. Future studies are needed to understand the clinical impacts of these changes.
